# The impact of age-friendly communities on the quality of life of older adults based on structural equation modeling

**DOI:** 10.3389/fpubh.2025.1646195

**Published:** 2025-10-27

**Authors:** Ying Tang, Jie Xu, Jingyu Yu

**Affiliations:** ^1^School of Management, Hefei University of Technology, Hefei, China; ^2^College of Civil Engineering, Hefei University of Technology, Hefei, China

**Keywords:** age-friendly community, older adults, quality of life, structural equation modeling, healthcare

## Abstract

**Background:**

With the aging of China’s population deepening, the development of age-friendly communities (AFCs) is playing an important role in alleviating the pressure of older adult care and enhancing the quality of life (QoL) of older adults. This paper aims to investigate the complicated impact of AFCs on the QoL of older adults through a questionnaire survey.

**Methods:**

A questionnaire survey was designed to explore the intricate relationships between components of AFCs and QoL factors from older adults’ perspective. A total of 1,396 older adults completed the survey. Factor analysis was used to identify AFC components and QoL factors, and the relationships between AFC components and QoL factors were predicted using structural equation modeling (SEM).

**Results:**

Four AFC components and three QoL factors for older adults were identified. The SEM results revealed that (1) physical health was mainly positively influenced by green spaces and negatively affected by public healthcare services; (2) apart from public transportation, most AFC components were indicative of psychological health; and (3) social networks were primarily influenced by green spaces and community support.

**Conclusion:**

To provide a better living environment for older adults, it is suggested that communities renovate existing structures to expand green spaces, add accessible facilities, and increase greenery. Communities should also enhance social environments by improving healthcare services, ensuring accessible medical care, and organizing cultural and recreational activities to promote older adults’ social participation and improve their QoL.

## 1 Introduction

In the global context, the aging population phenomenon is gaining increasing significance. According to World Population Prospects 2024 published by the United Nations, it is projected that by the late 2070s, the global population of individuals aged 65 and above will reach 2.2 billion, surpassing the number of individuals under 18 years old (1). Developed countries, such as Japan and Germany, are already experiencing heavily aging societies, while developing countries also face challenges caused by accelerating aging rates. Since entering the aging society stage in 2000, China has witnessed a deepening level of population aging. At the end of 2023, there were 297 million people aged 60 or above in China, accounting for 21.1% of the country’s total population (2). Compared to developed countries, China has transitioned from an aging society to a deeply aging society at a faster pace. To effectively address the challenges posed by population aging, the Chinese government has initiated efforts to establish age-friendly communities (AFCs) since 2020.

AFCs, which were first proposed and promoted by the World Health Organization (WHO), focus on meeting the needs of older people and improving their quality of life (QoL) and wellbeing (3). According to recent data, the WHO Global Network for Age-friendly Cities and Communities currently includes 1,606 cities and communities in 53 countries, covering over 330 million people worldwide (4); only 39 of these cities and communities are in China (including 18 in Hong Kong) (5). Different countries have formulated different frameworks and guidelines for AFCs (6). In the United States, the contents of AFC construction include housing planning, health and wellbeing, accessible services, and community participation (7). The United Kingdom promotes the concept of Lifetime Neighborhoods, which includes housing, free public transportation, a green environment, community safety, culture and learning opportunities, and care and support services (8). Canada was one of the first countries to propose the concept of healthy communities and start building age-friendly healthy communities (9); it primarily evaluates the degree of age-friendliness in terms of physical environment, social environment, and service in order to determine indicators suitable for rural and remote communities that meet older people’s needs while enhancing community support for their health and active living. The Japanese government actively advocates for aging-in-place by promoting home adaptations for older populations and constructing small-scale multifunctional nursing facilities in familiar neighborhoods, with the fundamental goal of providing support (10). Hence, it is evident that no universally applicable standard for the development of AFCs exists, necessitating the need for tailored solutions based on local contexts.

In recent years, the Chinese government has actively promoted the construction of AFCs. However, not every component of AFCs proposed by the WHO is suitable for the development of AFCs in China (11). In promoting the construction of AFCs, China primarily focuses on six aspects: living environment, transportation, community services, social participation, cultural life, and technological services (12). Taking into account the framework for AFCs proposed by the WHO and other countries, outdoor space and architecture, public transport, housing, social participation, community support, and health services are shared priorities in most countries. This article will discuss the impact of AFCs on the QoL of older adults, focusing on four aspects: green spaces, public health services, public transportation, and social support. The reason for not considering housing and social participation in this context is that this article takes a community-oriented approach, which focuses on the core public services and infrastructure of the community’s daily functions. This focus enables us to accurately examine the impact of these aspects managed by the community on the lives of the older adult. Therefore, the current study discusses the impact of different components of AFCs on older adults’ QoL across four dimensions. The objectives of this study are to (1) identify the components of AFCs, (2) ascertain the QoL factors of older adults, (3) examine the impact of AFC components on QoL factors for older adults, and (4) propose strategies to enhance the QoL of older adults and optimize the development of AFCs. The study aims to explore the development of AFCs in China, supplementing and enriching the existing literature on AFCs and the QoL of older adults. It investigates the impact of different components of AFCs on the QoL of older adults in China and provides empirical recommendations.

## 2 Literature review

With the acceleration of global aging, many countries and regions have begun practicing AFC construction, their efforts resulting in certain achievements. In the United Kingdom, London adopts an inclusive design strategy to ensure a friendly environment for older people, while Manchester, the first city in the UK to join the WHO Global Network of AFCs, encourages retrofitting housing and public places through policymaking and funding support (13). The United States provides policy and regulatory support for developing AFCs while encouraging research institutions and enterprises to develop specialized products that meet older people’s special needs (14). Japan focuses on using smart devices, such as smart home systems and wearable health monitoring devices, to protect older people’s safety while also establishing community safety networks to ensure their living security (15). Japan is the country in Asia that entered the aging society stage earliest. It has been promoting the construction of ‘longevity communities’ and advocating for the establishment of a comprehensive older adult care model that combines healthcare and nursing services within large community settings (16). Although different countries have different focuses with regard to AFCs, they all aim to improve older people’s QoL in various ways. Table 1 shows the emphasis of AFC construction in five countries.

**Table 1:** The emphasis of AFC construction in five countries.

	UK	US	Japan	Canada	China
Outdoor space and architecture	√		√	√	√
Transportation	√	√	√	√	√
Housing	√	√	√	√	
Social participation		√	√	√	√
Respect and tolerance	√			√	
Communication and information	√		√	√	
Community support and health services	√	√	√	√	√
Civic engagement and employment				√	

The concept of “Lifetime Neighborhoods” was introduced in the UK in 2007. In the United States, the American Association of Retired Persons (AARP) published The Livable Communities Evaluation Guide in 2005. In Canada, Age-Friendly Rural and Remote Communities: A Guide was published in 2007. The establishment of longevity communities has been promoted in Japan. The Notice on the Implementation of Demonstrative National Older-Friendly Community Creation Work was proposed by China in 2020.

QoL for older adults is generally considered to be a multidimensional concept. The WHO divides QoL into eight main dimensions: outdoor spaces, public transportation, housing, social participation, respect and social inclusion, communication and information, community, and health services (17). The main QoL factors for older adults have been identified as physical health, mental health, and social networks (18). Physical health refers to the physical fitness, pain management, and daily activity ability of older adults. Being in good physical condition is the foundation for maintaining a high QoL (19, 20) Psychological health reflects the satisfaction and happiness in older adults’ lives and effectively prevents and alleviates symptoms of depression (21, 22). Social networks provide emotional support and a sense of belonging for older adults, allowing them to enjoy richer life experiences through interactions with family, friends, and the community (23). Although existing research has explored the factors influencing QoL for older adults, further investigation is needed into the specific components of AFCs and their impact on older adults’ QoL. Therefore, it is important to further discuss the influence of each component of AFCs on the QoL of older adults in order to optimize AFC development and enhance their living environment.

The existing research has shown that the establishment of AFCs can effectively enhance the QoL of older adults from different dimensions (24–26). In recent years, the construction of AFCs has gradually attracted attention in various parts of China, enhancing older adults’ QoL by improving both their material and social environment. Local governments are also proposing policies to create suitable and livable environments for older adults in rural areas, thereby promoting their social participation (27).

Green spaces are important places for older adults to socialize and engage in outdoor activities, providing fitness equipment and entertainment facilities suitable for them, which promotes their physical health and enhances their life satisfaction (28, 29).

Public healthcare service institutions can provide basic medical services for older adults. The level of improvement in community public healthcare services has a significant promoting effect on older adults’ wellbeing. Providing psychological healthcare for older adults can effectively alleviate negative emotions (30, 31). With continuous technological advancements, intelligent older adult care services will become a future trend in older adult care (32).

Public transportation provides convenient travel options for older adults, and its accessible facilities reduce the difficulties of getting around, enabling them to participate in more social activities (33). Additionally, the fixed routes and schedules of public transportation also help older people to better plan their trips.

Community support is particularly important in enhancing the overall QoL of older adults (34). Moreover, such support can enhance older adults’ satisfaction with their lives (35). Dikken et al. (36) developed a specific questionnaire to gain a better understanding of older individuals’ experiences regarding the age-friendliness of cities. The composition of AFCs is therefore assumed to determine the QoL of older individuals. Previous studies on the impact of AFCs on QoL factors are summarized in Table 2.

**Table 2:** Literature research on the impact of AFCs on QoL among older adults.

AFC	Impact on QoL	Source
Green spaces	Improving the neighborhood environment can improve the sense of community identity and self-health evaluation of older adults.	(50)
	A safe, adequate, and barrier-free living environment can help older adults adapt to age-related changes and positively influence their well-being; otherwise, they may experience maladjustment, and their subjective well-being could be impaired.	(37)
	Ensuring access to ramps to enter buildings, disseminating information about the accessibility of parks and buildings, and social or community events may reduce older adults’ perceived disconnectedness.	(48)
	Investment in neighborhood social environments is expected to benefit the well-being of older citizens, especially as dependence on these environments increases with age.	(51)
Public healthcare services	Improving the quality of services has become the key to enhancing older adults’ satisfaction in the community.	(38)
	Community care services result in a significant improvement in both the objective and subjective health and well-being of older adults.	(52)
	Community and health services are significantly associated with life satisfaction for the young-old group but not the old-old.	(53)
Public transportation	Increasing the overall walkability of urban and suburban areas, with multiple destinations within short distances, can support more walking, less driving, and healthier aging for older Americans.	(54)
	Transportation has been found to significantly reduce depressive symptoms in rural neighborhoods.	(21)
	Transportation inclusion can help enhance the health, social participation, and subjective well-being of older adults.	(33)
Community support	Fostering interactions through community events is important for enhancing older adults’ feelings of connectedness.	(49)
	Strengthening community social service programs and improving the built environment can reduce social disconnection and loneliness among older adults, ultimately enhancing their life satisfaction.	(39)

**Figure 1:**
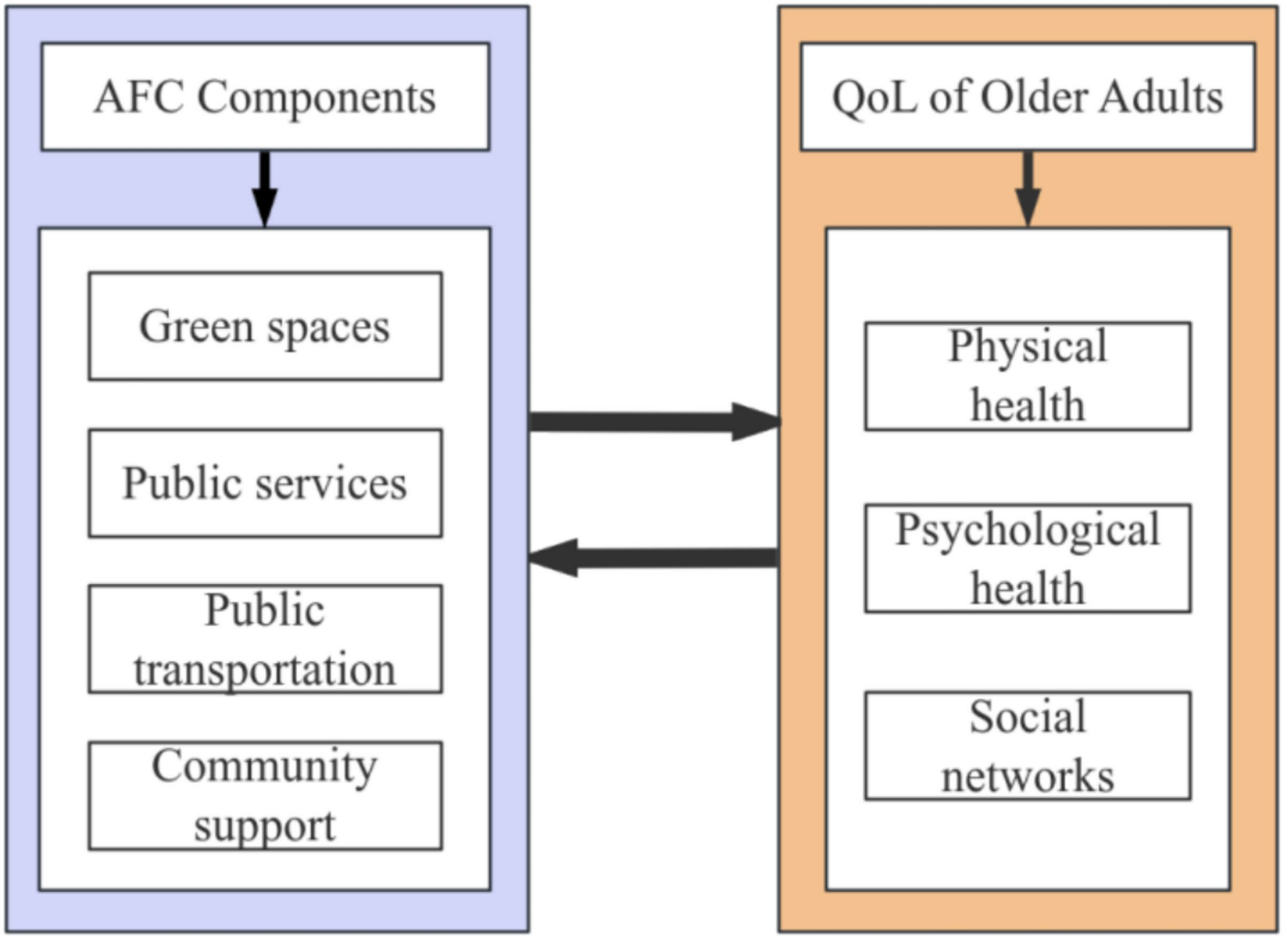
Conceptual model of the impact of AFC components on the domains of older adults’ QoL.

Previous studies have examined the relationships between several AFC components and the QoL of older adults. Green spaces enhance the happiness and community identity of the older adult by optimizing the community environment (37). Public healthcare services directly affects community satisfaction (38). The accessibility of public transportation can alleviate depressive symptoms and contribute to improved social participation among the older adult (21). Community support effectively reduces feelings of loneliness and enhances life satisfaction by strengthening social activities and services (39). However, there is limited research on the comprehensive impact of AFC systems on various aspects of older adults’ QoL. In fact, AFCs are comprehensive systems with different elements, such as green spaces, community support, healthcare, and so on. Therefore, it is important to consider AFCs as a whole and to systematically evaluate the different components of AFCs to explore their multifaceted effects on older adults’ QoL. The conceptual model for this study is depicted in Figure 1.

## 3 Methodology

A questionnaire survey was conducted to investigate the impact of AFCs on the QoL of older adults. The questionnaire is shown in the appendix. The questionnaire was divided into three main sections: Appendix A, which covered the background information of the respondents (including age, gender, education level, living arrangements, and health status); Appendix B, which assessed the respondents’ satisfaction with current AFCs (green spaces, public healthcare services, public transportation, and community support); and Appendix C, which focused on their QoL (physical health, mental health, and social networks). The questionnaire incorporated items from the WHO Age-Friendly Cities Checklist and the WHOQOL-BREF scale to measure AFC components and QoL, respectively. The abbreviated version of the WHOQOL-BREF was chosen to mitigate respondent fatigue among the older adult (40). We conducted a questionnaire survey in Hefei, China, in 2022 to investigate the complicated relationships between the components of AFCs and the QoL of older adults. In 2022, the permanent population of Hefei was 9.634 million, and the older adult population aged 60 and above was 1.516 million, accounting for 15.74%. The study received support from the local community, including logistical support and assistance with participant recruitment.

The study employed a purposive sampling method in order to ensure data validity. Older respondents aged 60 or above who had resided in various communities for at least 6 months were selected to guarantee their sufficient understanding of AFCs. Furthermore, face-to-face interviews were conducted by trained research assistants, who provided support and explanations whenever necessary in order to ensure the accuracy and comprehensibility of the questionnaire responses. A total of 1,383 older adults completed the survey; 44% of the respondents were male and 56% were female. With regard to age there were 50.6% of participants in age group 60–67, 37% in age group 70–79, 11.4% in age group 80–89 and only 1% in age group over 90 years of age. Over 80% of the respondents were not well educated, and only 4.1% had attended college. Almost half of the respondents lived with their husband or wife, 13.7% lived alone, 42.1% lived with their children, and 5.0% lived with their relatives. Over 60% of the respondents were healthy; 12.2% were generally healthy and 0.9% were generally unhealthy.

The collected data underwent multiple statistical methods to construct a comprehensive structural model of the intricate relationship between different components of AFCs and the QoL of older adults. First, exploratory factor analysis (EFA) and confirmatory factor analysis (CFA) were conducted to identify AFC factors and QoL factors for adults. The analysis was conducted using SPSS Statistics 27. EFA was utilized to preliminarily identify latent factors by examining the correlation matrix of the survey items and determining suitable data for factor extraction. Subsequently, CFA was employed to validate the initially identified factor structure by confirming the significant loadings of all items on their hypothesized latent factors, ensuring the validity and reliability of the model. To further establish internal consistency, a reliability analysis was performed on the identified factors using Cronbach’s alpha coefficient.

A structural equation model was adopted to incorporate the measurement items and latent factors. Structural equation modeling (SEM) allows the estimation of unobservable latent variables and the examination of complex causal relationships among these variables. The structural equation model was constructed to examine the relationships between the latent variables of AFC components and QoL factors. The SEM framework consists of two components: the measurement model and the structural model.

The measurement model was first established to link observed variables to their respective latent factors (C1-C4 and Q1-Q3) through confirmatory factor analysis, as shown in Equations 1, 2.


(1) $$X=\Lambda_{x}\xi +\delta$$

(2) $$Y=\Lambda_{y}\eta +\varepsilon$$

where X and Y are vectors of observed indicators for AFC components and QoL factors, respectively; ξ and η denote the latent constructs; $\Lambda_{x}$ and $\Lambda_{y}$ are the matrices of standardized factor loadings; δ and ϵ represent measurement errors.

The structural model then specified the causal paths between AFC components and QoL domains, as shown in Equation 3.


(3) η=Bη+Γξ+ζ

Where η represents the endogenous latent variables, ξ denotes the exogenous latent variables, B and Γ are matrices of structural coefficients, and ζ denotes the residual errors.

The fit indices used for evaluating model adequacy included root mean square error approximation, comparative fit index, normed fit index, incremental fit index, and non-normed fit index. The model with the optimal-fit indices was selected for interpretation. The analysis was conducted using AMOS 28, which facilitates the graphical representation of complex models and the handling of latent variables with multiple indicators.

## 4 Results

Factor analysis was adopted to reduce a large set of items to a smaller set of meaningful factors. To further validate the internal consistency, reliability analysis was conducted on the identified factors. The results of the EFA of AFCs and the QoL of older adults are presented in Tables 3, 4. The Kaiser–Meyer–Oklin (KMO) values for the components of AFCs and the factors of older adults’ QoL were 0.771 and 0.708, respectively, both of which exceeded the recommended value of 0.6. Bartlett’s Test reached statistical significance, thus supporting the factorability of the correlation matrix.

**Table 3:** Results of factor analysis for age-friendly communities.

Factors	Items	Description	Factor loading	Alpha ( α )
C1 Green spaces	afc1	There are clean and comfortable public spaces in the community.	0.755	0.8535
	afc2	Pedestrian walkways within the community are sufficient for wheelchair access.	0.745	
	afc3	There are sufficient accessible facilities (elevators, slopes, and anti-slip floor tiles).	0.766	
	afc4	There are sufficient activity venues in the community.	0.687	
	afc5	Sufficient intersection passage time around the community	0.68	
	afc6	There are sufficient outdoor lighting and security rooms in the community to ensure community safety.	0.569	
C2 Public healthcare services	afc7	Community healthcare facilities and services within walking distance of older adults	0.63	0.8438
	afc8	Community health service centers provide convenient medical services.	0.613	
	afc9	Hospitals around the community have good accessibility.	0.748	
	afc10	Hospitals around the community can provide suitable healthcare services for older adults.	0.722	
C3 Public transportation	afc11	Reasonable public transportation costs	0.582	0.8711
	afc12	The public transportation network around the community is developed, making travel convenient.	0.708	
	afc13	Reliable and punctual public transportation around the community	0.9	
	afc14	Public transportation should be clean and equipped with priority seats.	0.88	
	afc15	Public transportation stations are set up within walking distance of older adults, with rest seats and rain shelters.	0.75	
	afc16	Complete and clear public transportation information	0.67	
	afc17	Community and surrounding public activity venues can be reached on foot or by public transportation.	0.339	
C4 Community support	afc18	Older adults are often asked how their needs are met.	0.743	0.8095
	afc19	Regular communication between older people and community workers.	0.794	
	afc20	Community-based telephone services are clearly expressed and more patient toward older adults.	0.731	
	afc21	Community older adult care facilities are safe and regularly maintained.	0.595	
	afc22	There are universities for older adults around the community.	0.697	
	afc23	Public activities organized specifically for older adults in the community	0.715	

**Table 4:** Results of factor analysis for quality of life of older adults.

Factors	Items	Description	Factor loading	Alpha ( α )
Q1 Physical health	qol1	Are you satisfied with your health condition?	0.824	0.881
	qol2	Do you have any physical discomfort that prevents you from doing anything?	0.797	
	qol3	Do you have enough energy to cope with daily life?	0.706	
	qol4	How about your mobility?	0.748	
	qol5	Are you satisfied with your sleep?	0.785
Q2 Psychological health	qol6	Do you find life enjoyable?	0.569	0.9148
	qol7	Can you concentrate your attention?	0.803
	qol8	Do you feel safe in daily life?	0.786
	qol9	Are you satisfied with your appearance?	0.863
	qol10	Are you satisfied with your ability to do daily tasks?	0.814
	qol11	Do you have negative emotions?	0.833
Q3 Social networks	qol12	Do you think life is meaningful?	0.832	0.9147
	qol13	Do you have enough money?	0.843
	qol14	Are you satisfied with the support you receive from your friends?	0.741
	qol15	Are you satisfied with your living conditions?	0.776
	qol16	Are you satisfied with the healthcare services you have received?	0.821
	qol17	Are you satisfied with your transportation situation?	0.789

The four AFC components [i.e., green spaces (C1), public healthcare services (C2), public transportation (C3), and community support (C4)] had coefficient alphas ranging from 0.809 to 0.885. Apart from item afc17, all items were appropriately loaded into acceptable factors. Item afc17 was removed from public transportation (C3) due to its low factor loading of 0.339. Reliability testing showed a significant improvement in the Cronbach’s alpha value for public transportation (C3) after removing item afc17. As older adults in China age, their physical functions gradually decline and long walks or using public transportation may impose additional burdens on their bodies. Considering safety, older adults prefer to engage in daily activities near their homes or within the community. Additionally, the social circles of older adults are relatively small and mainly concentrated in the community and nearby areas, which also makes them more inclined to participate in activities in these familiar places.

Three QoL factors were identified: physical health (Q1), psychological health (Q2), and social networks (Q3). Each of these QoL factors had a coefficient alpha >0.8, which meant that the factors were reliable, internally consistent, and valid for further analysis. Physical health includes health condition, absence of physical discomfort, energy levels for daily life, mobility, and satisfaction with sleep. Psychological health emphasizes enjoying life, ability to concentrate, feeling safe in daily life, and satisfaction with appearance and ability to perform daily tasks, as well as the absence of negative emotions. The social networks factor includes enjoying life, achieving financial sufficiency, and being satisfied with support from friends, living conditions, healthcare services received, and transportation situation.

To investigate the relationship between AFC components and the QoL of older adults, the SEM was further developed using AMOS. Through SEM, unobservable latent variables of the AFC factors and QoL factors were estimated from observed indicators by establishing the relationships (paths) among the latent variables (41). SEM can also be used to investigate complicated causal relationships using both quantitative data and qualitative assumptions (42).

**Figure 2:**
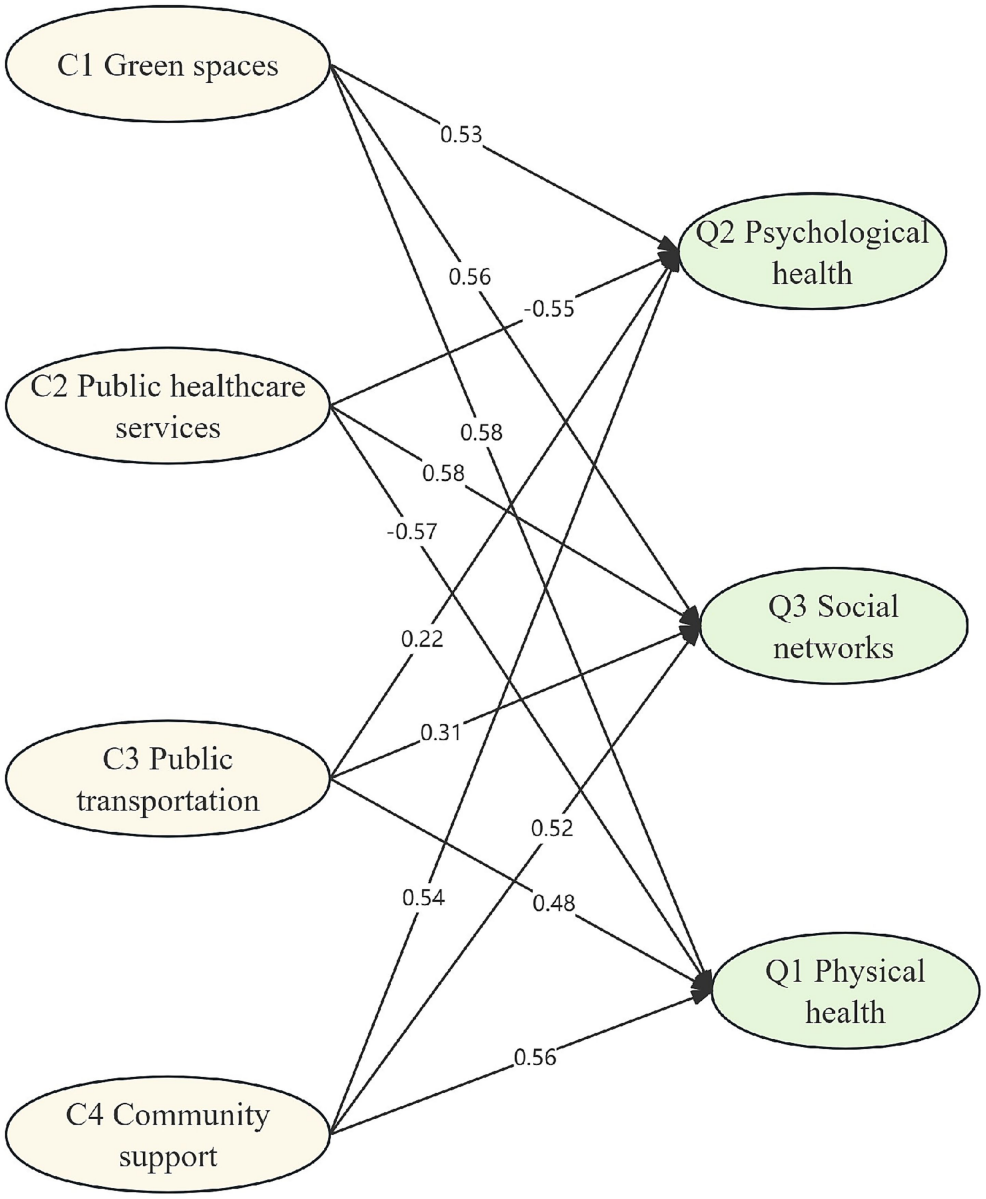
Structural relationships between AFCs and QoL for older adults.

The measurement model was first evaluated using confirmatory factor analysis to verify the relationships between the latent constructs and their observed indicators. The standardized factor loadings of each indicator are presented in Table 5. The paths among the AFC and QoL factors in the structural model are shown in Figure 2. The hypothesized model was tested and subsequently refined to improve model fit. The results revealed that this final model had the lowest badness-of-fit indices (RMSEA: 0.073) and the highest overall fit indices (CFI: 0.883, NFI: 0.831, IFI: 0.894, NNFI: 0.819) (43). The paths of the SEM indicated the following: (1) green spaces (C1) positively influenced physical health (Q1), psychological health (Q2) and social networks (Q3); (2) public healthcare services (C2) negatively affected physical health (Q1) and psychological health (Q2); (3) the relationships between public transportation (C3) and the QoL factors were not significant; and (4) community support (C4) impacted psychological health (Q2) and social networks (Q3) positively.

**Table 5:** Results of measurement model.

Latent variable	Observed variable	Standardized factor loading
C1 Green spaces	afc1	0.64
	afc2	0.83
	afc3	0.84
	afc4	0.56
	afc5	0.59
	afc6	0.48
C2 Public healthcare services	afc7	0.58
	afc8	0.71
	afc9	0.64
	afc10	0.59
C3 Public transportation	afc11	0.75
	afc12	0.67
	afc13	0.57
	afc14	0.70
	afc15	0.91
	afc16	0.89
	afc17	0.74
C4 Community support	afc18	0.66
	afc19	0.31
	afc20	0.67
	afc21	0.69
	afc22	0.63
	afc23	0.55
Q1 Physical health	qol12	0.91
	qol13	0.92
	qol14	0.90
	qol15	0.88
	qol16	0.93
	qol17	0.93
Q2 Psychological health	qol1	0.93
	qol2	0.92
	qol3	0.87
	qol4	0.89
	qol5	0.92
Q3 Social networks	qol6	0.90
	qol7	0.89
	qol8	0.93
	qol9	0.91
	qol10	0.92
	qol11	0.82

## 5 Conclusion

The results support the argument that green spaces play a crucial role in significantly influencing the physical health, mental wellbeing, and social networks of older adults, which aligns with previous studies (44, 45). Green spaces, equipped with convenient facilities such as good lighting, spacious sidewalks, and comfortable resting areas, provide suitable venues for activities like daily walks, tai chi, and square dancing (40). These activities not only enhance physical fitness and prevent chronic diseases but also stimulate the vitality of older adults. Open spaces such as parks within the community can help improve the mood and reduce feelings of loneliness among older individuals, which is beneficial for their mental health. Moreover, green spaces and recreational areas in the community provide more social opportunities to meet with friends and neighbors, helping to strengthen the social network of older people and increase their emotional connection and sense of belonging in society. Therefore, communities can enhance older adults’ QoL by regularly maintaining and upgrading accessible facilities while improving environmental hygiene management to optimize green spaces further.

It is interesting to note that public healthcare services have a negative impact on the physical and psychological health of older individuals. Although community healthcare institutions may offer convenient and nearby medical services for older adults, the quality of their medical services often fails to meet older adults’ needs. Community healthcare facilities often lack advanced medical equipment and specialized medical personnel, making it difficult to provide complex or emergency medical services. Many older adults tend to go to tertiary hospitals with more comprehensive facilities and higher medical standards, especially in China (46). However, the long waiting times in tertiary hospitals affect their trust in and reliance on medical services, as well as causing inconvenience and psychological pressure when accessing necessary healthcare services. Thus, neither community hospitals nor top-tier hospitals provide adequate mental health treatment services for older adults, and this has a negative influence on their physical and psychological health.

The research findings indicate that not all components of AFCs have an impact on the QoL of older adults. In this study, public transportation did not have a significant impact on QoL factors, possibly because older adults prefer to engage in activities near their community and reduce their reliance on public transportation. Additionally, with the implementation of the “15-min living circle” concept in many cities in China, the daily needs of residents can be met within a 15-min walking or cycling distance. In this case, older adults may be more inclined to participate in activities within their community rather than relying solely on public transportation for travel, making the impact of public transportation on QoL negligible (47).

Community support has a significant positive impact on the mental health and social networks of older individuals. Regular communication between older adults and community workers, along with attention to their needs by the community, makes them feel cared for and supported, thus reducing feelings of loneliness and depression, which can improve their mental wellbeing (48). A supportive environment within the community, coupled with various social activities, provides more opportunities for social interaction among older adults. This helps to enhance emotional connections and foster a sense of belonging among them, ultimately improving their overall QoL (49). Therefore, communities should strengthen their focus on responding to the needs of older individuals by organizing regular community activities suitable for them while enhancing training for community workers to increase sensitivity toward these needs. Furthermore, improving the quality and safety of community older adult care facilities is crucial in ensuring comfortable living conditions that are secure.

## 6 Recommendations

### 6.1 Policy and practical implications

The results of the current study demonstrate the integrated relationships between AFC components and the QoL of older adults. Below are some suggestions for improving the construction of communities that are friendly to older individuals. Green space is crucial for the construction of AFCs. To enhance the physical environment of old neighborhoods, it is suggested that school facilities should be utilized and old buildings should be renovated to expand green spaces. Furthermore, accessible facilities, such as handrails and seating areas, should be installed; greenery and shade should be increased; and fitness equipment and recreational facilities suitable for older adults should be added in order to create aging-friendly transformations in outdoor areas. Encouraging active participation in outdoor activities among older adults helps meet their specific needs.

To enable public healthcare services to better fulfill their role in supporting older adults’ QoL, communities should enhance the coverage and quality of public facilities and services, improve the community healthcare service system, upgrade medical services in community health centers, and ensure timely access to medical care for older people. Therefore, it is necessary for us to enhance the quality of community health services and improve the accessibility of tertiary hospitals, particularly in terms of medical services and psychological health treatments. Communities can also utilize internet technology to establish smart older adult care service platforms that offer convenient services such as remote medical care, emergency rescue support, housekeeping appointments, and so on. Additionally, attention should be given to addressing mental health issues among older individuals by providing psychological counseling and support services. Simultaneously, establishing community canteens or meal assistance points to provide balanced meals and organizing various cultural and recreational activities, such as tai chi practice, square dancing, choir singing, and so on, can meet the basic living needs and spiritual-cultural pursuits of older people while enhancing their QoL.

### 6.2 Research implications

Although this study has explored the impact of AFCs on the QoL of older people in detail through SEM, there are still some limitations. First, the sample size of the questionnaire survey was limited, and although it included 1,396 older adults, the geographical and socioeconomic backgrounds of the sample may affect the universality of the results. Second, the study mainly used quantitative questionnaire methods, which provide broad data support but may overlook subjective experiences and individual differences among older people. Additionally, the study did not incorporate the potential role of digitalization, such as smart technologies and digital community platforms, which are increasingly relevant to modern age-friendly communities.

To address these limitations, future research should aim for more diverse samples from various geographic and socioeconomic backgrounds to enhance the generalizability and representativeness of the results. Combining quantitative and qualitative methods can allow the comprehensive exploration of AFCs’ impact on older people’s QoL to gain a more complete understanding. Longitudinal studies can track changes in older people’s QoL over different time periods to analyze the long-term effects resulting from the construction of AFCs. Future studies should also examine how digital tools, such as health monitoring wearables and smart home systems, could be integrated as components of AFCs or as moderating factors influencing older adults’ QoL. The implementation effect of related policies for AFCs can also be studied by analyzing their actual impact on older people’s QoL; specific community case studies could summarize successful experiences as well as practical lessons learned that provide actionable guidance for policymaking or community building programs.

## 7 Conclusion

Facing the challenge of rapid population aging in China, the Chinese government is actively promoting the construction of AFCs. However, there are currently only a limited number of AFCs in China that meet the requirements set by the WHO. Despite significant efforts from the government in terms of policies and resources, there is still a lack of systematic research on the effectiveness and specific impact mechanisms of these communities in practical operation. Therefore, this study sought to explore the impact of various components of AFCs on the QoL of older individuals through a large-scale questionnaire survey. The research findings identified four components of AFCs (green spaces, public healthcare services, public transportation, and community support) and three domains of older adults’ QoL (physical health, psychological health, and social networks). The results from the structural equation model revealed the comprehensive relationship between AFC components and older adults’ QoL. The SEM results predicted the comprehensive relationship between AFC components and older adults’ QoL. The study found that physical health QoL is mainly positively influenced by green spaces but negatively affected by public healthcare services. Except for public transportation, most components of AFCs can indicate psychological QoL. Social networks QoL is primarily influenced by green spaces and community support.

On the basis of the current research findings, suggestions have been proposed for improving AFCs by expanding green spaces, improving public healthcare services, enhancing healthcare services, and organizing various recreational and social activities. Strengthening community support is also recommended for promoting communication between older adults and community workers as well as improving the quality and safety of older adult care facilities in the community. Future research should consider increasing sample size and geographical diversity to enhance the generalizability of results. Additionally, employing digital methods, such as remote monitoring and analyzing engagement with smart community platforms, can provide objective, longitudinal data on older adults’ behaviors and needs, thereby validating and enriching the findings of this study.

## Data Availability

The original contributions presented in the study are included in the article/Supplementary material, further inquiries can be directed to the corresponding author.
