# Supplementary material for: The impact of age-friendly communities on the quality of life of older adults based on structural equation modeling
Source: Front Public Health. 2025 Oct 27;13:1646195. doi: 10.3389/fpubh.2025.1646195 (PMC12613276; doi:10.3389/fpubh.2025.1646195)
Supplement: Supplementary file 1 [file Table_1.docx]

**Appendix A**

**Basic Information**

| Basic Information | | | | | |
| --- | --- | --- | --- | --- | --- |
| Age |  | Gender |  | Ethnicity |  |
| Number of children |  | Educational attainment |  | Occupation |  |
| Construction age |  | Resident population |  | Number of floors where one lives |  |
| House type |  | Living style | 1- Children 2- spouse 3- Living alone | Area |  |

| Health status | | | |
| --- | --- | --- | --- |
| Physical condition | 1- Healthy 2- Generally healthy 3- Average 4- Generally unhealthy | Be able to take care of oneself | 1 Yes 2 No |
| Frequency of hospital visits  (Except for sudden illnesses) | 1- Once a month 2- once every three months 3- once every six months 4- once a year 5-____ | | |

**Appendix B**

**Satisfaction with elderly-friendly living environment (WHO aging-friendly cities checklist)**

|  | **Age-friendly cities** | **Satisfaction** | | | | |
| --- | --- | --- | --- | --- | --- | --- |
|  |  | **Very dissatisfied** | **Not very satisfied** | **Average** | **Quite satisfied** | **Very satisfied** |
| 1 | There are clean and comfortable public spaces in the community. |  |  |  |  |  |
| 2 | Pedestrian walkways within the community are sufficient for wheelchair access. |  |  |  |  |  |
| 3 | There are sufficient accessible facilities (elevators, slopes, and anti-slip floor tiles). |  |  |  |  |  |
| 4 | There are sufficient activity venues in the community. |  |  |  |  |  |
| 5 | Sufficient intersection passage time around the community |  |  |  |  |  |
| 6 | There are sufficient outdoor lighting and security rooms in the community to ensure community safety. |  |  |  |  |  |
| 7 | Community healthcare facilities and services within walking distance of older adults |  |  |  |  |  |
| 8 | Community health service centers provide convenient medical services. |  |  |  |  |  |
| 9 | Hospitals around the community have good accessibility. |  |  |  |  |  |
| 10 | Hospitals around the community can provide suitable healthcare services for older adults. |  |  |  |  |  |
| 11 | Reasonable public transportation costs |  |  |  |  |  |
| 12 | The public transportation network around the community is developed, making travel convenient. |  |  |  |  |  |
| 13 | Reliable and punctual public transportation around the community |  |  |  |  |  |
| 14 | Public transportation should be clean and equipped with priority seats. |  |  |  |  |  |
| 15 | Public transportation stations are set up within walking distance of older adults, with rest seats and rain shelters. |  |  |  |  |  |
| 16 | Complete and clear public transportation information |  |  |  |  |  |
| 17 | Community and surrounding public activity venues can be reached on foot or by public transportation. |  |  |  |  |  |
| 18 | Older adults are often asked how their needs are met. |  |  |  |  |  |
| 19 | Regular communication between older people and community workers. |  |  |  |  |  |
| 20 | Community-based telephone services are clearly expressed and more patient towards older adults. |  |  |  |  |  |
| 21 | Community older adult care facilities are safe and regularly maintained. |  |  |  |  |  |
| 22 | There are universities for older adults around the community. |  |  |  |  |  |
| 23 | Public activities organized specifically for older adults in the community |  |  |  |  |  |

**Appendix C**

**Health and Quality of Life of the Elderly (WHOQoL-BREF)**

| Are you satisfied with your health condition? | A. Very dissatisfied | B. Not satisfied | C. Average | D. Satisfied | E. Very satisfied |
| --- | --- | --- | --- | --- | --- |
| Do you have any physical discomfort that prevents you from doing anything? | A. Often | B. Sometimes | C. Average | D. Rarely | E. None |
| Do you find life enjoyable? | A. No | B. Very few | C. Average | D. Yes | E. Very much |
| Do you think life is meaningful? | A. No | B. Very few | C. Average | D. Yes | E. Very much |
| Can you concentrate your attention? | A. No | B. Rarely | C. Average | D. Most of the time | E. Totally able |
| Do you feel safe in your daily life? | A. Very unsafe | B. Unsafe | C. Average | D. Safety | E. Very safe |
| Do you have enough energy to cope with daily life? | A. No | B. Very little | C. Average | D. Yes | E. Very much |
| Are you satisfied with your appearance? | A. Very dissatisfied | B. Dissatisfied | C. Average | D. Satisfied | E. Very satisfied |
| Do you have enough money? | A. Not quite enough | B. Not enough | C. Average | D. Enough | E. Quite enough |
| How about your mobility? | A. Very poor | B. Poor | C. Average | D. Good | E. Very good |
| Are you satisfied with your sleep? | A. Very dissatisfied | B. Not satisfied | C. Average | D. Satisfied | E. Very satisfied |
| Are you satisfied with your ability to do daily tasks? | A. Very dissatisfied | B. Not satisfied | C. Average | D. Satisfied | E. Very satisfied |
| Are you satisfied with the support you received from your friends? | A. Very dissatisfied | B. Not satisfied | C. Average | D. Satisfied | E. Very satisfied |
| Are you satisfied with your living conditions? | A. Very dissatisfied | B. Not satisfied | C. Average | D. Satisfied | E. Very satisfied |
| Are you satisfied with the health care services you received? | A. Very dissatisfied | B. Not satisfied | C. Average | D. Satisfied | E. Very satisfied |
| Are you satisfied with your traffic situation? | A. Very dissatisfied | B. Not satisfied | C. Average | D. Satisfied | E. Very satisfied |
| Do you have negative emotions? | A. Always | B. Often | C. Average | D. Occasionally | E. None |
